# Cervical cerclage outcomes by indication and cervical length across gestational-age thresholds: a historical cohort study

**DOI:** 10.1186/s12884-026-09595-z

**Published:** 2026-07-04

**Authors:** Aybekcan Batman, Muhammed Kutluhan Azman, Hale Özer Çaltek, Ali Konu, Ecem Okşen, Alper Türkoğlu, Hakan Erenel

**Affiliations:** 1 Department of Obstetrics and Gynecology, Division of Perinatology, Başakşehir Çam and Sakura City Hospital, Istanbul, Turkey; 2 Department of Obstetrics and Gynecology, Başakşehir Çam and Sakura City Hospital, Istanbul, Turkey; 3 Present address: Department of Obstetrics and Gynecology, Kırkağaç State Hospital, Manisa, Turkey

**Keywords:** Cervical cerclage, Preterm birth, Uterine cervical incompetence, Cervical length measurement, Cohort studies, Gestational age

## Abstract

**Background:**

Cervical cerclage reduces the risk of preterm birth, but factors associated with its success may not be uniform. Among singleton pregnancies with ultrasound- or physical examination-indicated cerclage (no history-based indication), we examined whether factors associated with delivery differ by gestational-age threshold.

**Methods:**

This single-center historical cohort included 114 women undergoing cerclage between May 2020 and December 2025: ultrasound-indicated with cervical length 10 to < 20 mm (Group 1, *n* = 41) or < 10 mm (Group 2, *n* = 32), and physical examination-indicated cervical dilatation (Group 3, *n* = 41). All cerclages used the McDonald technique with polypropylene monofilament suture. The primary outcome was gestational age at delivery, assessed at ≥ 34, < 32, and < 28 weeks. Firth penalized multivariable logistic regression evaluated study group, maternal age, and gestational age at cerclage as primary covariates; post-cerclage cervical length was analyzed separately as a secondary post-treatment-adjusted variable. All models were exploratory, and p values were nominal.

**Results:**

In these exploratory models, factors associated with delivery differed across thresholds. For delivery ≥ 34 weeks, gestational age at cerclage was the only covariate independently associated with the outcome (aOR 1.28 per week, 95% CI 1.04 to 1.59; *p* = 0.021). For delivery before 32 weeks (aOR 2.96, 95% CI 1.08 to 8.66; *p* = 0.034) and before 28 weeks (aOR 9.36, 95% CI 1.98 to 91.11; *p* = 0.003), physical examination-indicated cerclage was the only covariate that reached significance; 11 of the 14 deliveries before 28 weeks occurred in this group, so this estimate is imprecise and near-separating. The numerically higher rate of delivery ≥ 34 weeks in Group 2 than Group 1 (78.1% vs. 70.7%) was not statistically significant. Apparent discrimination was moderate (AUC 0.69 to 0.77), without internal validation.

**Conclusions:**

In this exploratory single-center cohort, the factors associated with delivery varied with the gestational-age threshold examined. Near term, later gestational age at cerclage was associated with delivery ≥ 34 weeks; at earlier thresholds, physical examination-indicated cerclage identified a higher-risk group for very and extreme preterm delivery, although these estimates were imprecise and partly confounded by indication severity and co-interventions. Findings should be interpreted as hypothesis-generating and require confirmation in larger prospective studies.

**Supplementary Information:**

The online version contains supplementary material available at https://doi.org/10.1186/s12884-026-09595-z.

## Background

Preterm birth, defined as delivery before 37 weeks of gestation, remains the leading cause of neonatal mortality and long-term morbidity worldwide [1]. In 2020, an estimated 13.4 million infants, 9.9% of all live births — were born preterm globally [2]. The clinical impact of prematurity increases with decreasing gestational age, with higher rates of neonatal mortality and severe morbidities — such as bronchopulmonary dysplasia, intraventricular hemorrhage, and necrotizing enterocolitis — particularly below 32 and 28 weeks [3, 4].

Cervical insufficiency refers to the inability of the cervix to retain a pregnancy in the second trimester in the absence of uterine contractions [5]. The pathogenesis is multifactorial and may involve congenital cervical anomalies, prior cervical trauma from surgical procedures such as conization or dilatation and curettage, and inflammatory or microbial mechanisms causing premature cervical remodeling [6, 7]. Cervical insufficiency may be detected by obstetric history, transvaginal cervical length (CL) measurement, or physical examination [8]. A mid-trimester CL of ≤ 25 mm defines a short cervix [9].

Cervical cerclage is the primary surgical intervention for cervical insufficiency. Depending on the timing and clinical findings, cerclage is classified as history-indicated, ultrasound-indicated, or physical examination-indicated (emergency or rescue) [10]. While cerclage reduces the risk of preterm birth in women at high risk [11], the management of women presenting with progressive cervical shortening or advanced cervical dilation in the second trimester remains a clinical challenge [6]. A recent meta-analysis demonstrated that emergency cerclage substantially reduces the rate of preterm birth before 28 weeks (38.2% vs. 93.0%) [8] and before 34 weeks (51% vs. 91%) [5] compared to expectant management. However, the success of cerclage depends on the degree of cervical shortening and the presence of cervical dilation [12, 13].

Although cervical dilation has been reported as an independent risk factor for cerclage failure [12, 14], studies often compare cerclage with expectant management [8] rather than directly between the indication groups. Previous studies have compared ultrasound- and physical examination-indicated cerclages as broad categories [6, 15], and others have stratified ultrasound-indicated cerclages by CL [16]. However, to our knowledge no prior study has directly compared these stratified subgroups with physical examination-indicated cerclage within a non-history-indicated singleton cohort.

We therefore evaluated cerclage outcomes in three indication strata: Ultrasound-indicated with CL 10 to < 20 mm, ultrasound-indicated with CL < 10 mm, and physical examination-indicated with cervical dilation at three gestational-age thresholds (≥ 34, < 32, and < 28 weeks).

## Methods

### Study design and setting

This historical cohort study was conducted at Başakşehir Çam and Sakura City Hospital between May 2020 and December 2025.

### Participants

During the study period, 130 women underwent cervical cerclage at our institution. Sixteen were excluded (12 with multiple gestations and 4 with a history of spontaneous preterm birth), leaving 114 singleton pregnancies for analysis. No formal sample-size calculation was performed; all eligible women undergoing cervical cerclage during the study period were included.

Eligibility required a singleton pregnancy with cerclage placement between 14 and 24 weeks for either a short cervix (< 20 mm on transvaginal ultrasound) or cervical dilatation on physical examination, complete clinical and obstetric records, and a delivery outcome documented at the same institution. Additional exclusions were fetal anomaly, premature rupture of membranes at cerclage, active vaginal bleeding, or clinical chorioamnionitis. Gestational age was established from the last menstrual period and confirmed by first-trimester ultrasound; where the two differed, first-trimester ultrasound was used. In pregnancies conceived through assisted reproduction, gestational age was based on the date of embryo transfer.

### Group definitions

Women were classified into three groups based on the indication for cerclage and pre-cerclage cervical findings. Group 1 (ultrasound-indicated, *n* = 41) included women with a transvaginal CL of 10 to < 20 mm. Group 2 (ultrasound-indicated, *n* = 32) included women with a transvaginal CL < 10 mm. Group 3 (physical examination-indicated, *n* = 41) included women with cervical dilatation ≥ 1 cm identified on speculum or digital examination, regardless of CL.

This three-group classification was based on the prognostic relevance of CL thresholds (10 mm [12] and 20 mm [17, 18]) reported in previous studies and on the established clinical distinction between ultrasound-indicated and physical examination-indicated cerclage [15]. This stratification separated progressively severe cervical pathology while keeping each group adequately sized.

### Cervical assessment

Transvaginal CL measurements were performed by experienced sonographers using a standardized technique [19]. The woman was placed in the dorsal lithotomy position with an empty bladder. A transvaginal probe was inserted into the anterior fornix, and the shortest CL was recorded after observing the cervix for at least three minutes. The shortest of three measurements was used for analysis. In Group 3, cervical dilatation was assessed by speculum examination, and by digital examination when indicated.

### Cerclage technique

All cerclage procedures used the high McDonald technique [20] with polypropylene (Prolene) suture, under regional or general anesthesia. To enable placement at the highest possible level on the cervix, a purse-string suture was placed at four to five anchor points as close to the internal os as feasible, while avoiding the uterine vessels. In women with prolapsed or visible membranes, a stepwise membrane reduction sequence was applied before suture placement: steep Trendelenburg positioning; gentle reduction with an inflated Foley catheter balloon when needed; transabdominal amnioreduction when membrane bulging persisted. Cerclage was placed once the membranes were repositioned above the level of the internal os.

### Peri-operative management

Women in the cervical dilatation group (Group 3) received a standardized peri-operative protocol consisting of intravenous ceftriaxone 1 g every 12 h, metronidazole 500 mg every 8 h, and oral azithromycin 1 g once daily for 48 h; rectal indomethacin 100 mg every 12 h for 48 h; and oral amoxicillin-clavulanate 1 g every 12 h from postoperative day 3 through day 7. Women in Groups 1 and 2 did not routinely receive antibiotic or tocolytic prophylaxis.

### Progesterone protocol

Women with a transvaginal CL of < 25 mm received daily vaginal micronized progesterone 200 mg until 36 weeks of gestation. In women with progressive cervical shortening or dilatation despite progesterone therapy, cerclage was placed with continuation of progesterone post-operatively [21]. Synthetic 17α-hydroxyprogesterone caproate was not used, as the PROLONG trial demonstrated no efficacy in preventing recurrent preterm birth [22].

## Outcomes

The primary outcome was gestational age at delivery, analyzed both as a continuous descriptive endpoint and at three predefined clinical thresholds (≥ 34, < 32, and < 28 weeks) for threshold-specific models. The three thresholds were defined a priori based on established clinical categories of preterm birth: moderate-to-late, very, and extreme preterm delivery, respectively [2]. These cutoffs are widely applied in cerclage outcome studies [5, 23]. Delivery at ≥ 34 weeks was treated as a favorable outcome, as neonatal outcomes are generally favorable beyond this gestation, whereas delivery before 32 and before 28 weeks represented increasingly adverse outcomes. Secondary outcomes were pregnancy latency (interval from cerclage to delivery), birthweight, low birthweight (< 2500 g), very low birthweight (< 1500 g) [17], Apgar scores < 7 at 1 and 5 min, and live birth.

### Statistical analysis

Analyses of baseline, procedural, and outcome variables were carried out in SPSS version 26.0 (IBM Corp., Armonk, NY, USA). Continuous variables were summarized as mean (SD) or median with interquartile range, depending on their distribution; categorical variables were reported as number and percentage. Normality was checked using the Shapiro-Wilk test. For comparisons across the three study groups, one-way analysis of variance or the Kruskal–Wallis test was used for continuous variables, and the chi-square test or Fisher’s exact test for categorical variables, as appropriate. The Bonferroni adjustment was applied to post hoc pairwise comparisons. No women in the analytic cohort had missing data for variables used in the multivariable models. Statistical significance was set at a two-sided p-value < 0.05.

Multivariable modeling and graphical analyses were conducted in R version 4.6.0 (R Foundation for Statistical Computing, Vienna, Austria). Gestational age at delivery was displayed across the three study groups using a box plot generated with the *ggplot2* and *ggsignif* packages. To evaluate independent factors associated with delivery at ≥ 34, <32, and < 28 weeks of gestation, we fitted multivariable logistic regression models using Firth’s penalized likelihood method with the *logistf* package. The Firth method was selected a priori because the small number of events at the earlier thresholds (29 events at < 32 weeks and 14 at < 28 weeks, of which 11 occurred in the physical examination-indicated group) made maximum-likelihood estimation prone to separation and bias [24]. Because the study was not powered for formal interaction testing or confirmatory threshold-specific prediction, all multivariable models were considered exploratory, and the p values from the threshold-specific models were interpreted as nominal and were not adjusted for the number of outcomes, thresholds, or covariates examined. The primary models included study group, maternal age, and gestational age at cerclage placement as prespecified clinically relevant covariates. Post-cerclage cervical length was measured after the intervention and may lie on the causal pathway between indication, anatomical restoration, and delivery outcome; including it alongside the group variable can over-adjust the group comparison. For this reason, models excluding post-cerclage cervical length were specified as the primary analyses, and models additionally including post-cerclage cervical length were presented as secondary, post-treatment-adjusted analyses (Additional file A3). Nulliparity was evaluated as an additional candidate covariate but was not retained because it did not improve model fit and was not associated with any outcome in preliminary models. Adjusted odds ratios with 95% profile likelihood confidence intervals are reported. Model discrimination was summarized by the area under the receiver operating characteristic curve (AUC) and its 95% confidence interval (R package *pROC*; Fig. 3). These values represent apparent discrimination within the development sample; no internal validation or optimism correction was performed, and they should be interpreted as optimistic. Calibration was assessed with the Hosmer–Lemeshow goodness-of-fit test, results of which are provided in Additional file A2. Variance inflation factors did not indicate multicollinearity (Additional file A1).

## Results

### Study cohort and baseline characteristics

**Fig. 1:**
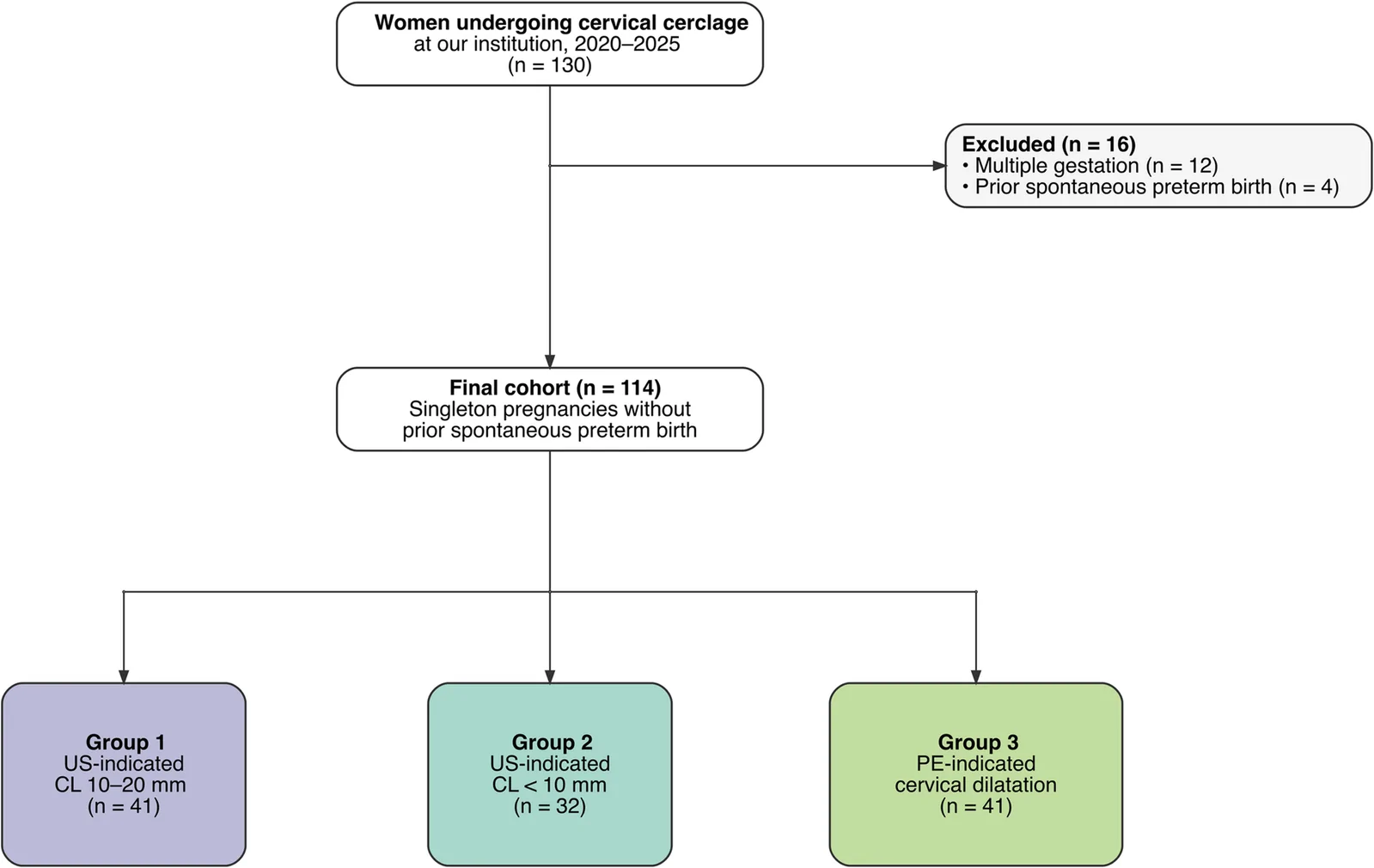
Patient flow through the study. Of 130 women undergoing cervical cerclage at our institution between 2020 and 2025, 16 were excluded (12 multiple gestations and 4 with prior spontaneous preterm birth). The final cohort of 114 singleton pregnancies was stratified into three groups by cerclage indication

Of 130 women undergoing cervical cerclage during the study period, 114 singleton pregnancies remained after exclusions (Fig. 1). Baseline maternal characteristics are presented in Table 1. Maternal age differed across groups, being highest in Group 1 (*p* = 0.037); other baseline characteristics were comparable.

**Table 1:** Baseline maternal characteristics by study group

Variables	Group 1 CL 10 to < 20 mm (*N* = 41)	Group 2 CL < 10 mm (*N* = 32)	Group 3 Cervical dilatation (*N* = 41)	*p* value
Maternal age (years)	31.3 (5.9)	28.7 (5.4)	28.0 (6.5)	**0.037**
Gravida, median (IQR)	2 (1–3)	1.5 (1–2)	2 (1–2)	0.141
Parity, median (IQR)	0 (0–1)	0 (0–0)	0 (0–1)	0.077
Nulliparity, n (%)	24 (58.5)	26 (81.2)	30 (73.2)	0.095
Parity 1, n (%)	10 (24.4)	5 (15.6)	7 (17.1)	0.580
Parity ≥ 2, n (%)	7 (17.1)	1 (3.1)	4 (9.8)	0.153
IVF/ICSI conception, n (%)	9 (22.0)	5 (15.6)	5 (12.2)	0.487
History of spontaneous abortion, n (%)	19 (46.3)	11 (34.4)	16 (39.0)	0.572

Values are presented as mean (SD), median (IQR), or n (%), as appropriate. A p value < 0.05 was considered statistically significant

*Abbreviations*: *CL* Cervical length, *IVF/ICSI* In vitro fertilization/intracytoplasmic sperm injection

### Cervical findings and cerclage characteristics

Pre-cerclage CL and procedural details are summarized in Table 2. In Group 3, pre-cerclage CL could not be measured due to cervical dilatation. Gestational age at cerclage placement and vaginal progesterone use did not differ between groups (*p* = 0.133 and *p* = 0.852, respectively). Post-cerclage CL differed between groups, being highest in Group 1 (*p* = 0.028).

**Table 2:** Pregnancy and neonatal outcomes

Variables	Group 1 CL 10 to < 20 mm (*N* = 41)	Group 2 CL < 10 mm (*N* = 32)	Group 3 Cervical dilatation (*N* = 41)	*p* value
Pre-cerclage cervical length, mm	13.8 (3.2)	5.4 (3.0)	—	**< 0.001**
Use of vaginal progesterone, n (%)	20 (48.8)	16 (50.0)	18 (43.9)	0.852
GA at cerclage placement (weeks)	21.9 (2.2)	22.4 (1.5)	21.5 (2.0)	0.133
GA at delivery (weeks)	34.4 (3.3)	35.3 (4.3)	32.0 (5.9)	**0.007** ^**a**^
Pregnancy latency (weeks)	12.5 (3.2)	12.8 (4.5)	10.4 (5.9)	0.060
Delivery at ≥ 34 weeks, n (%)	29 (70.7)	25 (78.1)	21 (51.2)	**0.039**
Delivery < 32 weeks, n (%)	7 (17.1)	5 (15.6)	17 (41.5)	**0.013**
Delivery < 28 weeks, n (%)	1 (2.4)	2 (6.2)	11 (26.8)	**0.002** ^**b**^
Post-cerclage cervical length, mm	26.1 (5.7)	22.5 (7.7)	22.1 (8.0)	**0.028** ^**b**^
Apgar score < 7 at 1 min, n (%)	17 (41.5)	9 (28.1)	20 (48.8)	0.200
Apgar score < 7 at 5 min, n (%)	0 (0)	1 (3.1)	11 (26.8)	**< 0.001**

Values are presented as mean (SD) or n (%)

*Abbreviations*: *CL* Cervical length, *GA* Gestational age, *SD* Standard deviation

ᵃGroup 3 vs. Groups 1 and 2 (Bonferroni)

ᵇGroup 1 vs. Group 3 (Bonferroni)

### Pregnancy and delivery outcomes

Pregnancy outcomes are presented in Table 2. The mean gestational age at delivery was lowest in Group 3 (*p* = 0.007), whereas pregnancy latency did not differ between groups (*p* = 0.060). Delivery at ≥ 34 weeks was least frequent in Group 3 (*p* = 0.039), which also had the highest rates of preterm birth < 32 weeks (*p* = 0.013) and < 28 weeks (*p* = 0.002).

### Neonatal outcomes

Neonatal outcomes are summarized in Table 2. A 1-minute Apgar score of < 7 did not differ between groups (*p* = 0.200). A 5-minute Apgar score of < 7 was more frequent in Group 3 than in Groups 1 and 2 (*p* < 0.001).

### Subgroup analysis by post-cerclage cervical length

Delivery outcomes were also examined according to whether post-cerclage CL reached ≥ 20 mm (Table 3). In both ultrasound-indicated groups, women who achieved a post-cerclage CL ≥ 20 mm had numerically higher rates of delivery at ≥ 34 weeks than those who did not. This descriptive pattern was not apparent in Group 3.

**Table 3:** Delivery outcomes according to post-cerclage cervical length

Variables	Group 1 (*N* = 41)		Group 2 (*N* = 32)		Group 3 (*N* = 41)	
Post-cerclage CL	< 20 mm (*n* = 17)	≥ 20 mm (*n* = 24)	< 20 mm (*n* = 13)	≥ 20 mm (*n* = 19)	< 20 mm (*n* = 27)	≥ 20 mm (*n* = 14)
Delivery at ≥ 34 weeks, n (%)	11 (64.7)	18 (75.0)	8 (61.5)	17 (89.5)	13 (48.1)	8 (57.1)
Delivery < 32 weeks, n (%)	4 (23.5)	3 (12.5)	3 (23.1)	2 (10.5)	12 (44.4)	5 (35.7)
Delivery < 28 weeks, n (%)	0 (0)	1 (4.2)	1 (7.7)	1 (5.3)	8 (29.6)	3 (21.4)

Values are presented as n (%). No formal statistical comparison was performed for these subgroups owing to small cell sizes

*Abbreviations*: *CL* Cervical length

### Gestational age at delivery and factors associated with preterm birth

**Fig. 2:**
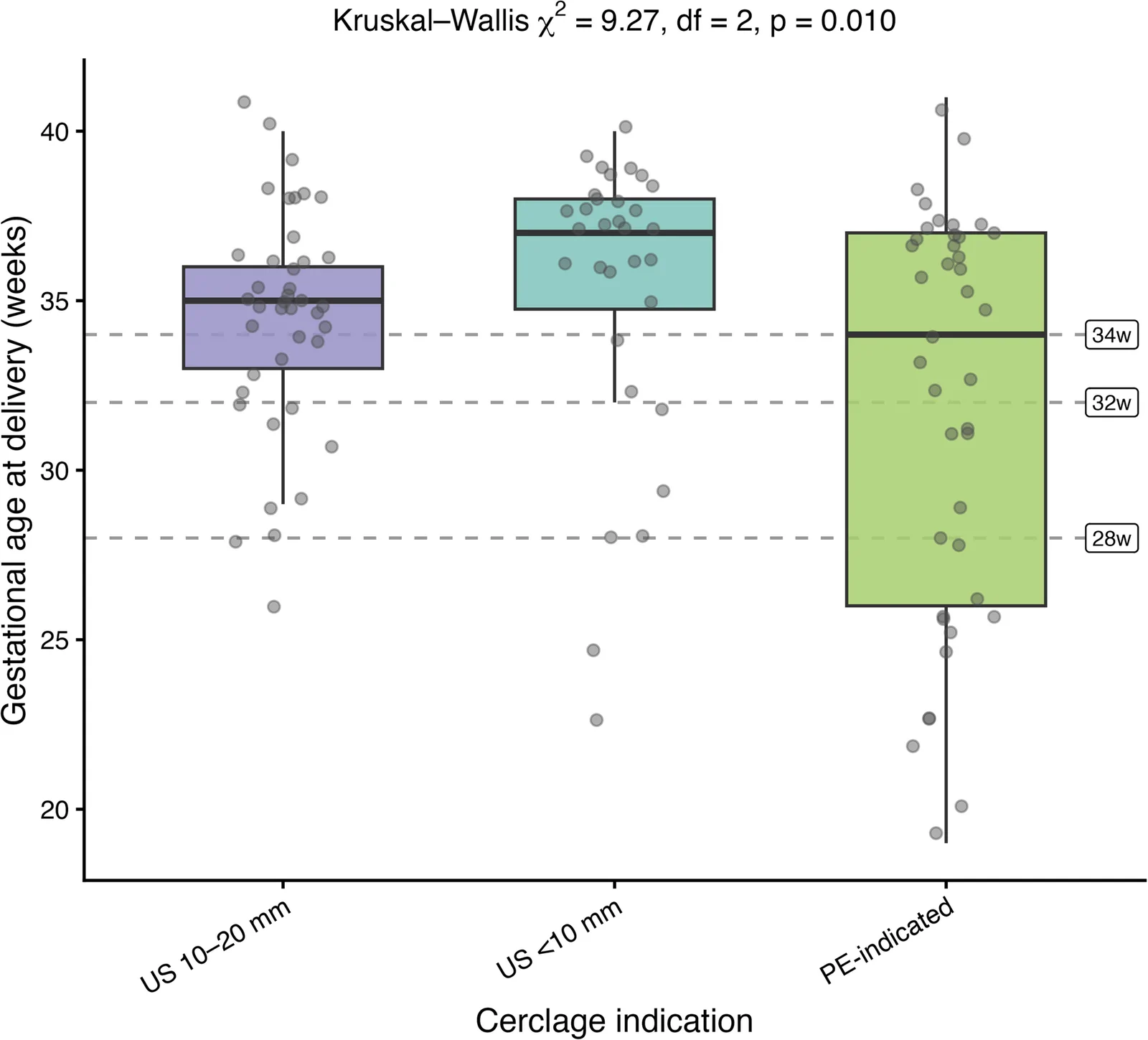
Box plot of gestational age at delivery across the three study groups. Group 1: ultrasound-indicated cerclage with cervical length of 10 to < 20 mm (n = 41); Group 2: ultrasound-indicated cerclage with cervical length < 10 mm (n = 32); Group 3: physical examination-indicated cerclage with cervical dilatation (n = 41). Boxes represent the interquartile range with the median indicated by the central line; whiskers extend to 1.5 × IQR; individual points represent each woman (jittered for visibility). Dashed horizontal lines mark clinically relevant gestational age thresholds at 28, 32, and 34 weeks. Group differences were assessed using the Kruskal–Wallis test (χ² = 9.27, df = 2, p = 0.010); pairwise group comparisons are reported in Table 2 (Bonferroni post-hoc test)

The distribution of gestational age at delivery differed significantly across the three groups (Kruskal–Wallis χ² = 9.27, df = 2, *p* = 0.010; Fig. 2), consistent with the parametric pairwise comparisons reported in Table 2.

**Fig. 3:**
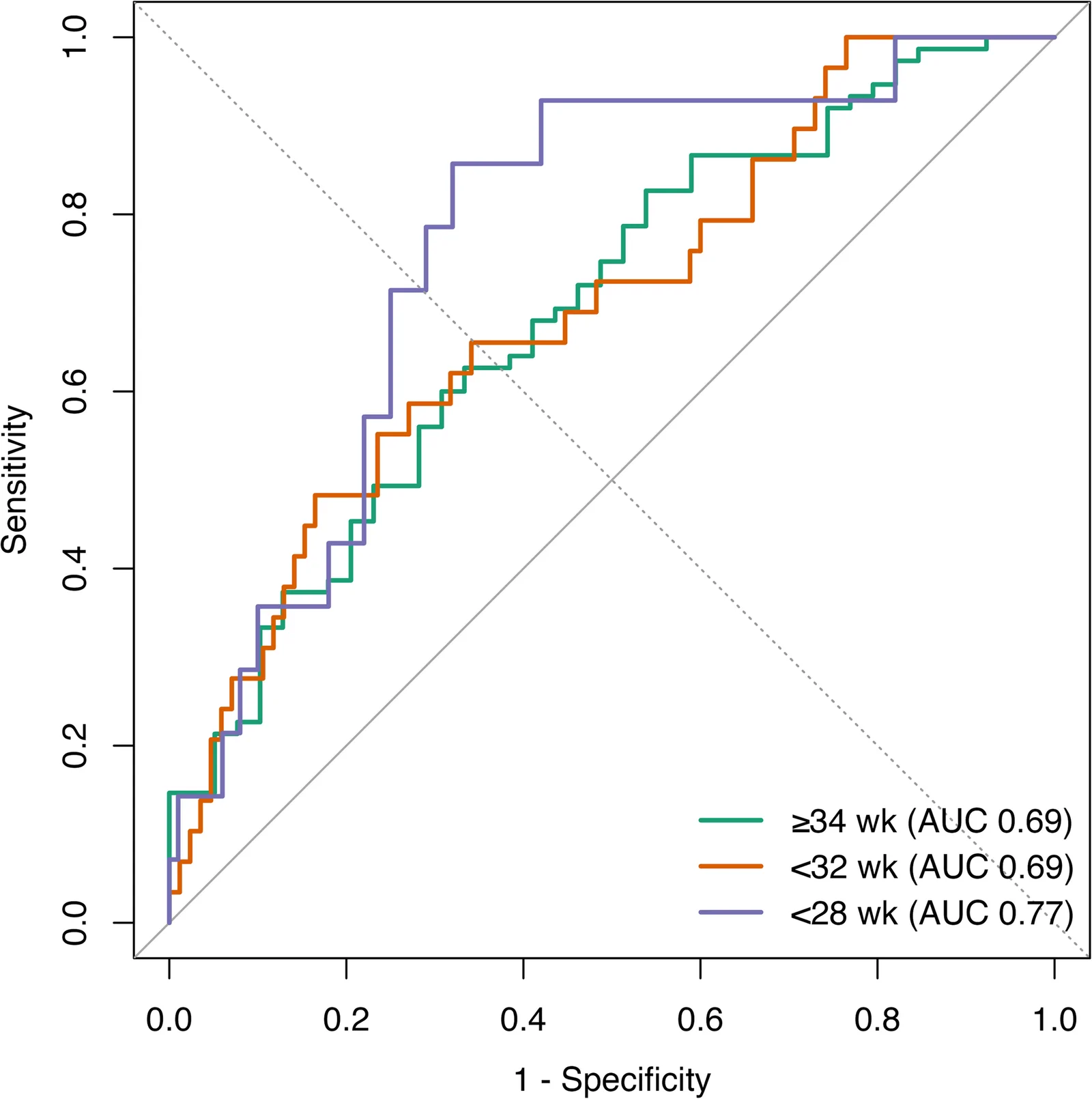
Receiver operating characteristic (ROC) curves for the three threshold-specific multivariable models (delivery at ≥34, <32, and <28 weeks of gestation). AUC, area under the curve

In the exploratory multivariable models, the factors associated with outcome differed across the three gestational-age thresholds (Table 4); given the limited number of events, these patterns are interpreted as hypothesis-generating rather than as demonstrated effect modification. At delivery ≥ 34 weeks, gestational age at cerclage was the only covariate independently associated with the outcome (aOR 1.28 per week, 95% CI 1.04 to 1.59; *p* = 0.021). For delivery before 32 weeks, physical examination-indicated cerclage (Group 3) was the only covariate that reached significance (aOR 2.96, 95% CI 1.08 to 8.66; *p* = 0.034). For delivery before 28 weeks, Group 3 again carried the only significant association, with a large but imprecise estimate (aOR 9.36, 95% CI 1.98 to 91.11; *p* = 0.003); 11 of the 14 deliveries before 28 weeks occurred in this group, so the model approached separation and the estimate should be read as reflecting that already-dilated women delivered extremely preterm rather than as a precise effect size. Maternal age was not associated with outcome at any threshold, and gestational age at cerclage was not retained as an independent factor at the earlier thresholds. Models that additionally included post-cerclage cervical length, presented as secondary post-treatment-adjusted analyses, are reported in the Additional file A3; in these models the association of Group 3 with delivery before 28 weeks was attenuated (aOR 8.11, 95% CI 1.63 to 80.62), consistent with over-adjustment by a post-intervention variable. Apparent model discrimination was moderate (area under the curve 0.69 for delivery ≥ 34 weeks, 0.69 for delivery before 32 weeks, and 0.77 for delivery before 28 weeks; Fig. 3); these values were not internally validated and are likely optimistic. No relevant multicollinearity was observed (Additional file A1).

**Table 4:** Factors associated with preterm birth at three gestational-age thresholds (primary models)

Covariate	aOR	95% CI	*p*
≥ 34 weeks (75 events)			
Group 2 (US < 10 mm) vs. Group 1	1.24	0.42 to 3.78	0.702
Group 3 (PE-indicated) vs. Group 1	0.47	0.18 to 1.20	0.115
Maternal age (per year)	1.00	0.93 to 1.07	0.968
GA at cerclage (per week)	**1.28**	**1.04 to 1.59**	**0.021**
*< 32 weeks (29 events)*			
Group 2 (US < 10 mm) vs. Group 1	0.96	0.27 to 3.30	0.953
Group 3 (PE-indicated) vs. Group 1	**2.96**	**1.08 to 8.66**	**0.034**
Maternal age (per year)	0.99	0.92 to 1.06	0.708
GA at cerclage (per week)	0.86	0.69 to 1.08	0.191
*< 28 weeks (14 events)*			
Group 2 (US < 10 mm) vs. Group 1	2.05	0.26 to 23.25	0.492
Group 3 (PE-indicated) vs. Group 1	**9.36**	**1.98 to 91.11**	**0.003**
Maternal age (per year)	0.98	0.89 to 1.08	0.746
GA at cerclage (per week)	1.02	0.76 to 1.40	0.894

Firth penalized logistic regression with profile-likelihood 95% confidence intervals. Primary models included study group, maternal age, and gestational age at cerclage; post-cerclage cervical length, a post-intervention variable, was excluded and analysed separately (Additional file A3). Bold denotes *p* < 0.05. Apparent discrimination (area under the curve): ≥ 34 weeks 0.69 (95% CI 0.59 to 0.79); < 32 weeks 0.69 (95% CI 0.58 to 0.81); < 28 weeks 0.77 (95% CI 0.65 to 0.89), without internal validation or optimism correction. P values are nominal and were not adjusted for multiple thresholds or covariates

*aOR* Adjusted odds ratio, *CI* Confidence interval, *GA* Gestational age, *PE* Physical examination, *US* Ultrasound

## Discussion

We examined factors associated with preterm birth after cervical cerclage across three indication categories and three gestational-age thresholds. The main observation was that these factors differed across thresholds. At gestations approaching term, the timing of cerclage (gestational age at placement) was associated with delivery ≥ 34 weeks, whereas at the earlier thresholds the indication category dominated: physical examination-indicated cerclage carried markedly higher odds of delivery before 32 and before 28 weeks, although these estimates were imprecise (before 28 weeks, aOR 9.36, 95% CI 1.98 to 91.11; *p* = 0.003). Because the models were exploratory and based on small numbers of events, these cross-threshold differences are hypothesis-generating and do not establish that the determinants of cerclage outcome genuinely change with gestational age. Which covariate reached significance is itself sensitive to the limited and unevenly distributed events. They suggest that baseline anatomical restoration alone may not fully determine cerclage outcome. Two observations from our cohort frame the interpretation that follows: the non-significant but numerically favorable outcomes of Group 2 (CL < 10 mm), and the extreme-preterm risk concentrated in Group 3 despite a mean pregnancy latency of 10.4 weeks.

Within this cohort, Group 3 had the lowest rate of delivery at ≥ 34 weeks and the highest rates of delivery before 32 and before 28 weeks. This within-cohort ranking should be distinguished from how the group compares with the published literature: although Group 3 fared worst among our three groups, its absolute outcomes fell within the favorable range of reported physical examination-indicated cerclage series. Cerclage prolonged pregnancy by a mean of 10.4 weeks, comparable to the 9.7 to 13.4 weeks reported across recent series [15, 25, 26], and matching the range Pereira et al. characterized as a ‘favorable prolongation’ [27]. This interval advanced delivery from the typical peri-viable presentation into the early-to-moderate preterm range, where neonatal survival improves with each additional week. The higher rate of a 5-minute Apgar score < 7 in Group 3 is most likely mediated by the earlier gestational age at delivery in this group rather than reflecting an independent effect of indication; the observed rate of 26.8% nonetheless fell within the favorable range relative to published cohorts (33.3% in Golbasi et al.) [25]. The Pilarski et al. meta-analysis of 4795 women undergoing emergency cerclage reported an overall pregnancy loss rate of 27.3%, approximately half that observed with expectant management (RR 0.48) [28]. Against these benchmarks, the residual extreme-preterm risk in Group 3 most likely reflects the advanced cervical process already present at presentation rather than a failure of mechanical restoration.

Group 2 (CL < 10 mm) achieved a numerically higher rate of delivery at ≥ 34 weeks than Group 1 (78.1% vs. 70.7%), contrary to the conventional gradient between cervical shortening and preterm birth. This difference was not statistically significant, and adjustment did not alter its direction (Group 2 aOR 1.24 vs. Group 1, 95% CI 0.42 to 3.78; *p* = 0.702). A parallel pattern was reported by Bigelow et al., in whom preterm birth before 36 weeks occurred in 18.8% of cervices at 0–9 mm versus 25.5% at 10–19 mm (*p* = 0.600) [16], whereas Cook et al. reported the opposite direction (31% before 34 weeks at ≤ 10 mm versus 16% at 21–25 mm; *p* = 0.110) [29]. This inconsistency suggests that cervical length at presentation does not, by itself, determine outcome once a cerclage is placed. One possible explanation is that the biological process driving the shortening, rather than its measured extent, governs the response to cerclage: shortening without active intra-amniotic inflammation may respond favorably to mechanical closure, whereas shortening driven by ongoing inflammation may progress despite it. Because inflammatory and biochemical markers were not measured, this explanation remains a hypothesis for future study. Selection effects may also contribute, since women reaching extreme shortening have already survived the earliest losses.

Group 3 received a standardized perioperative regimen of intravenous ceftriaxone and metronidazole, oral azithromycin, rectal indomethacin, and oral amoxicillin-clavulanate, directed at two recognized risks in physical examination-indicated cerclage: ascending intra-amniotic infection from exposed membranes [8] and the surgical inflammatory response. This regimen was not administered to the ultrasound-indicated groups, so the group comparison reflects a combined clinical pathway (indication, membrane exposure, and co-intervention) rather than the effect of indication alone. The antimicrobial component mirrors the regimen used by Oh et al., who reported resolution of intra-amniotic inflammation in 75% of patients and treatment success in 59% [30]. In Group 3, delivery at ≥ 34 weeks reached 51.2%, while delivery before 32 weeks remained at 41.5% and before 28 weeks at 26.8%; more than one in four neonates were still delivered before 28 weeks. That this risk persisted despite intensive co-treatment is consistent with an advanced cervical process at presentation that perioperative therapy did not fully offset, although the contribution of the regimen itself cannot be quantified in this design.

All cerclages were performed using the McDonald technique [20] with a polypropylene (Prolene) monofilament suture. The McDonald technique is the most commonly applied approach in randomized trials and large cohorts [8]. It is preferred in physical examination-indicated cases because the Shirodkar alternative requires dissection of the vaginocervical mucosa and a longer operative time [26]. Placing the suture as high as possible addresses the risk associated with distal placement: Cook et al. demonstrated that sutures placed in the distal 10 mm or distal half of a closed cervix more than doubled the risk of preterm birth before 37 weeks [29]. When membranes were prolapsed, we applied the stepwise membrane-reduction sequence described in the Methods. No iatrogenic membrane rupture occurred at cerclage placement, in keeping with the low rates reported by emergency cerclage cohorts that use similar techniques [31]. Suture material also influences outcomes. Kindinger et al. linked braided sutures to vaginal dysbiosis and higher rates of pregnancy loss (intrauterine death 15% vs. 5%; preterm birth 28% vs. 17%) [32]. In the C-STICH randomized trial, pregnancy loss did not differ between suture types (8.0% vs. 7.6%, RR 1.05), although the trial authors recommended monofilament sutures to reduce the risk of maternal sepsis and chorioamnionitis [33]. Evidence specific to polypropylene is lacking; because Prolene is a monofilament suture, its use is consistent with the infection-related rationale supporting monofilament material.

Cervical insufficiency is increasingly viewed less as a purely mechanical defect and more as a syndrome involving inflammation and biochemical cervical remodeling [34]. Vink and Mourad characterized premature cervical remodeling as an active biological process, arguing that the cervix is not a passive, purely collagenous structure [7]. This failure is often inflammatory: Oh et al. reported acute intra-amniotic inflammation (elevated IL-6 or MMP-8) in up to 81% of women with cervical insufficiency, with culture-proven intra-amniotic infection in 8 to 52%, and found that cerclage placed during active inflammation produced uniformly poor outcomes [30]. Two observations in our cohort are compatible with this model, though neither confirms it. In Group 3, achieving a post-cerclage cervical length ≥ 20 mm was not associated with better outcomes (57.1% vs. 48.1%), which would be consistent with advanced remodeling that mechanical closure cannot reverse; this subgroup comparison was a secondary, post-treatment analysis and is hypothesis-generating. In Group 2, outcomes were favorable despite extreme shortening, which may reflect a predominantly mechanical rather than inflammatory process. Consistent with the importance of inflammation, Masui et al. reported postoperative serum C-reactive protein at one week as the only independent factor associated with cerclage failure (OR 9.75; 95% CI 2.99 to 31.8) [35]. Because we did not measure inflammatory or remodeling markers, we cannot establish the underlying reason. One possibility, for future study, is that cerclage is more effective before the cervix has remodeled extensively than after.

These models were exploratory, with few events. The factors associated with delivery were not the same at each threshold. At delivery ≥ 34 weeks, gestational age at cerclage was the only covariate independently associated with the outcome (aOR 1.28 per week, 95% CI 1.04 to 1.59; *p* = 0.021). In the secondary post-treatment-adjusted model, post-cerclage cervical length approached significance (aOR 1.06 per mm; *p* = 0.061), paralleling Mountain et al.’s finding that post-cerclage cervical length is a stronger continuous correlate of spontaneous preterm birth before 34 weeks than pre-cerclage cervical length [36]. At the earlier thresholds, physical examination-indicated cerclage was the covariate most strongly associated with delivery, before 32 weeks (aOR 2.96) and before 28 weeks (aOR 9.36), although the latter estimate was imprecise. That gestational age at cerclage was associated with the ≥ 34-week outcome contrasts with some prior cohorts [16], and likely reflects that women requiring earlier intervention carry a higher baseline risk and differing underlying pathophysiology [37]. This observation should not be interpreted as a reason to delay cerclage: in women with a short cervix (< 25 mm) before 24 weeks, cerclage reduces preterm birth [23]. The association is instead prognostic, and the risk of delivery at a peri-viable gestational age remains a central concern in physical examination-indicated cerclage that should inform counseling and shared decision-making [5].

This study has several strengths. Surgical management was relatively uniform: all procedures used the high McDonald technique [20] with Prolene monofilament suture. Restricting the cohort to singleton pregnancies reduced confounding from uterine overdistension. The three prespecified indication strata allowed the distinct profile of Group 2 to be examined separately from the advanced dilatation profile of Group 3. Pre- and post-cerclage cervical length were systematically recorded, enabling the secondary analysis of the relationship between anatomical restoration and outcome. Outcomes were examined at three clinical thresholds (≥ 34, < 32, and < 28 weeks), and for the sparse outcome categories, particularly delivery before 28 weeks, Firth’s penalized likelihood was used as an appropriate method to reduce small-sample bias [24]. Clinically, no iatrogenic membrane rupture occurred during cerclage placement, indicating that careful case selection was accompanied by safe technical feasibility.

This study has several limitations. The main limitation is the historical, single-center design, which leaves the analysis vulnerable to selection and information bias; the findings require confirmation in prospective multicenter cohorts. Second, the indication categories conflate the clinical indication with a co-intervention: Group 3 received a standardized perioperative antibiotic and indomethacin regimen that the ultrasound-indicated groups did not, so the group effect reflects a combined management pathway rather than indication alone, and its contribution cannot be separated in this design. Because this co-intervention would, if anything, be expected to improve Group 3 outcomes, the true difference associated with advanced dilatation may be larger than observed. Third, post-cerclage cervical length is measured after the intervention and may lie on the causal pathway to delivery; we therefore excluded it from the primary models and report it only as a secondary, post-treatment-adjusted variable, but residual over-adjustment in those secondary models cannot be excluded. Fourth, the threshold-specific models were exploratory and the p values were nominal, unadjusted for the multiple thresholds and covariates examined; the cross-threshold differences are hypothesis-generating. Fifth, the analysis of delivery before 28 weeks rests on only 14 events, 11 of them in Group 3; although Firth’s penalized likelihood reduced small-sample bias, the confidence intervals were wide and the corresponding estimates are imprecise. Sixth, Group 3 was not stratified by the degree of cervical dilatation, because subdividing an event-limited group would yield unstable estimates; some prognostic information is therefore not captured. Finally, inflammatory and biochemical markers (intra-amniotic IL-6 and MMP-8, serum C-reactive protein, and the vaginal microbiome) were not measured, so the remodeling framework discussed above rests on indirect inference and should be tested directly in future studies. A direct comparison with expectant management was not feasible, as cerclage is the standard of care at our institution; the literature-level comparison from the Pilarski et al. meta-analysis supports the benefit of cerclage over conservative management [28]. Neonatal outcomes beyond Apgar score and birthweight were not analyzed, as these are strongly determined by gestational age at delivery.

## Conclusion

In this exploratory single-center cohort, the factors associated with preterm birth after cerclage varied with the gestational-age threshold examined. As gestation approached term, later gestational age at cerclage was associated with delivery at or beyond 34 weeks, whereas at the earlier thresholds physical examination-indicated cerclage identified women at markedly higher risk of very and extreme preterm delivery, although these estimates were imprecise and partly reflect indication severity and concurrent co-interventions. Within the ultrasound-indicated groups, extreme cervical shortening was not associated with worse near-term outcomes, a non-significant observation that nonetheless suggests cervical length at presentation does not, by itself, determine the response to cerclage. Together these findings indicate that anatomical measurement alone may not fully capture risk after cerclage and that the underlying biological state of the cervix may be an equally important determinant, a hypothesis that should be tested in prospective studies pairing cerclage outcomes with inflammatory and remodeling biomarkers. Because the analyses were exploratory and event-limited, the patterns reported here are hypothesis-generating and require confirmation in larger, prospective, multicenter cohorts.

## Supplementary Information

Supplementary Material 1.

Supplementary Material 2.

Supplementary Material 3.

## Data Availability

The datasets generated and/or analysed during the current study are not publicly available due to privacy and ethical restrictions but are available from the corresponding author on reasonable request.

## Abbreviations

aOR: Adjusted odds ratio
AUC: Area under the curve
CI: Confidence interval
CL: Cervical length
GA: Gestational age
IVF/ICSI: In vitro fertilization/intracytoplasmic sperm injection
