# Supplementary material for: Cervical cerclage outcomes by indication and cervical length across gestational-age thresholds: a historical cohort study
Source: BMC Pregnancy Childbirth. 2026 Jul 4;26:1060. doi: 10.1186/s12884-026-09595-z (PMC13613748; doi:10.1186/s12884-026-09595-z)
Supplement: Supplementary file 1 [file 12884_2026_9595_MOESM1_ESM.docx]

**Table A2. Model calibration (Hosmer–Lemeshow goodness-of-fit test) for the three primary models.**

| **Model** | **χ²** | **df** | ***p*** |
| --- | --- | --- | --- |
| ≥34 weeks | 2.03 | 8 | 0.98 |
| <32 weeks | 8.02 | 8 | 0.43 |
| <28 weeks^a^ | 3.19 | 3 | 0.36 |

Hosmer–Lemeshow goodness-of-fit test for the primary Firth penalized logistic regression models. A *p* value greater than 0.05 indicates no evidence of poor fit (adequate calibration). ^a^The <28-week model was assessed with 5 groups rather than 10 because of the small number of events (14); with so few events the test has limited power, and this result should be interpreted with caution. χ², chi-squared statistic; df, degrees of freedom.
