# Supplementary material for: Cervical cerclage outcomes by indication and cervical length across gestational-age thresholds: a historical cohort study
Source: BMC Pregnancy Childbirth. 2026 Jul 4;26:1060. doi: 10.1186/s12884-026-09595-z (PMC13613748; doi:10.1186/s12884-026-09595-z)
Supplement: Supplementary file 2 [file 12884_2026_9595_MOESM2_ESM.docx]

**Table A3. Secondary (post-treatment-adjusted) models including post-cerclage cervical length.**

| **Covariate** | **aOR** | **95% CI** | ***p*** |
| --- | --- | --- | --- |
|  | | | |
| ***≥34 weeks (75 events)*** | | | |
| Group 2 (US <10 mm) vs Group 1 | 1.62 | 0.52 to 5.33 | 0.405 |
| Group 3 (PE-indicated) vs Group 1 | 0.62 | 0.23 to 1.70 | 0.354 |
| Maternal age (per year) | 1.01 | 0.94 to 1.09 | 0.746 |
| **GA at cerclage (per week)** | **1.31** | **1.06 to 1.64** | **0.014** |
| Post-cerclage CL (per mm) | 1.06 | 1.00 to 1.13 | 0.061 |
| ***<32 weeks (29 events)*** | | | |
| Group 2 (US <10 mm) vs Group 1 | 0.74 | 0.19 to 2.64 | 0.642 |
| Group 3 (PE-indicated) vs Group 1 | 2.23 | 0.77 to 6.82 | 0.140 |
| Maternal age (per year) | 0.97 | 0.90 to 1.05 | 0.475 |
| GA at cerclage (per week) | 0.84 | 0.67 to 1.06 | 0.143 |
| Post-cerclage CL (per mm) | 0.95 | 0.88 to 1.01 | 0.076 |
| ***<28 weeks (14 events)*** | | | |
| Group 2 (US <10 mm) vs Group 1 | 1.84 | 0.23 to 21.26 | 0.563 |
| **Group 3 (PE-indicated) vs Group 1** | **8.11** | **1.63 to 80.62** | **0.008** |
| Maternal age (per year) | 0.98 | 0.88 to 1.07 | 0.637 |
| GA at cerclage (per week) | 1.01 | 0.75 to 1.39 | 0.931 |
| Post-cerclage CL (per mm) | 0.97 | 0.90 to 1.05 | 0.467 |
|  | | | |

*Firth penalized logistic regression with profile-likelihood 95% confidence intervals. Secondary models added post-cerclage cervical length to the primary covariates. Because post-cerclage cervical length is measured after the intervention and may lie on the causal pathway between indication and outcome, these estimates are interpreted as post-treatment-adjusted associations rather than total effects of indication group. Bold denotes p < 0.05. P values are nominal and were not adjusted for multiple thresholds or covariates. aOR, adjusted odds ratio; CI, confidence interval; GA, gestational age; PE, physical examination; US, ultrasound.*
