# Supplementary material for: Cervical cerclage outcomes by indication and cervical length across gestational-age thresholds: a historical cohort study
Source: BMC Pregnancy Childbirth. 2026 Jul 4;26:1060. doi: 10.1186/s12884-026-09595-z (PMC13613748; doi:10.1186/s12884-026-09595-z)
Supplement: Supplementary file 3 [file 12884_2026_9595_MOESM3_ESM.docx]

**Table A1. Multicollinearity assessment (variance inflation factors) for the three primary models.**

| **Covariate** | **GVIF** | **df** | **Adjusted GVIF** |
| --- | --- | --- | --- |
| Study group | 1.10 | 2 | 1.02 |
| Maternal age | 1.14 | 1 | 1.07 |
| GA at cerclage | 1.07 | 1 | 1.04 |

Generalized variance inflation factors (GVIF) from the primary Firth penalized logistic regression models. The adjusted GVIF (GVIF raised to the power 1/(2·df)) allows comparison across terms with differing degrees of freedom. All values are close to 1, indicating no meaningful multicollinearity (a common concern threshold is VIF > 5). Because the three threshold-specific models share the same covariate set, these values apply to all three. GVIF, generalized variance inflation factor; df, degrees of freedom; GA, gestational age.
